# Application of Direct Current Atmospheric Pressure Glow Microdischarge Generated in Contact with a Flowing Liquid Solution for Synthesis of Au-Ag Core-Shell Nanoparticles

**DOI:** 10.3390/ma9040268

**Published:** 2016-04-06

**Authors:** Anna Dzimitrowicz, Piotr Jamroz, Marcin Nyk, Pawel Pohl

**Affiliations:** 1 Department of Analytical Chemistry and Chemical Metallurgy, Faculty of Chemistry, Wroclaw University of Technology, Wybrzeze St. Wyspianskiego 27, 50-370 Wroclaw, Poland; piotr.jamroz@pwr.edu.pl (P.J.); pawel.pohl@pwr.edu.pl (P.P.); 2 Department of Advanced Materials Engineering and Modelling, Faculty of Chemistry, Wroclaw University of Technology, Wybrzeze Stanislawa Wyspianskiego 27, 50-370 Wroclaw, Poland; marcin.nyk@pwr.edu.pl

**Keywords:** core-shell nanoparticles, atmospheric pressure plasmas, nanoparticles characterization, nanoparticles properties, silver, gold, 61.46.-w Structure of nanoscale materials, 78.67.Bf Nanocrystals, nanoparticles, and nanoclusters, 81.07.-b Nanoscale materials and structures: fabrication and characterization

## Abstract

A direct current atmospheric pressure glow microdischarge (dc-μAPGD) generated between an Ar nozzle microjet and a flowing liquid was applied to produce Au-Ag core-shell nanoparticles (Au@AgCSNPs) in a continuous flow system. Firstly, operating dc-μAPGD with the flowing solution of the Au(III) ions as the cathode, the Au nanoparticles (AuNPs) core was produced. Next, to produce the core-shell nanostructures, the collected AuNPs solution was immediately mixed with an AgNO_3_ solution and passed through the system with the reversed polarity to fabricate the Ag nanoshell on the AuNPs core. The formation of Au@AgCSNPs was confirmed using ultraviolet-visible (UV-Vis) absorbance spectrophotometry, transmission electron microscopy (TEM), and energy-dispersive X-ray spectroscopy (EDS). Three localized surface plasmon resonance absorption bands with wavelengths centered at 372, 546, and 675 nm were observed in the UV-Vis spectrum of Au@AgCSNPs, confirming the reduction of both the Au(III) and Ag(I) ions. The right configuration of metals in Au@AgCSNPs was evidenced by TEM. The Au core diameter was 10.2 ± 2.0 nm, while the thickness of the Ag nanoshell was 5.8 ± 1.8 nm. The elemental composition of the bimetallic nanoparticles was also confirmed by EDS. It is possible to obtain 90 mL of a solution containing Au@AgCSNPs per hour using the applied microdischarge system.

## 1. Introduction

Over the last few years, a significant increase in the scientific interest of metallic structures of nanometric size has been observed. The special properties of such nanoparticles (NPs), particularly the high surface area to volume ratio, have resulted in the rapid development of their synthesis and characterization methods [1,2,3,4]. The metallic NPs synthesized by different methods are used in many important fields of science, such as medicine [5], biotechnology [6], biodiagnostics [7], and cosmetology [8]. Researchers have found that the bimetallic core-shell nanoparticles (CSNPs) have significantly improved magnetic [9,10], optical [11], catalytic [12,13,14], photoluminescence [15], and electronic properties [16], as compared to their single pure metallic forms [17]. In addition, it was established that the exceptional properties of CSNPs could be tailored by altering the ratio between their core and shell, or by changing the kind of the applied material [12,17].

The Au@AgCSNPs, being inorganic/inorganic CSNPs and consisting of an Au core and an Ag shell, have been acknowledged to display many unique optical [11,18], catalytic [19], oxidative [20], and biosensoring properties [21], particularly due to the properties of both noble metals nanostructures. The exceptional optical properties of Au@AgCSNPs are dependent on the configuration of the metal core and metal shell. In this case, the AuNPs constitute the templates for the Ag nanoshell growth. The shell of the Au@AgSCNPs gives rise to nanoparticles with the optical properties that are similar to those displayed by AgNPs, such as the *d-s* band gap in the UV region, making them useful in surface-enhanced Raman scattering (SERS) [18,22,23]. For example, Liu and Guyot-Sionnest [23] reported that an Ag nanoshell deposited on AuNPs exhibits an additional damping of the plasmon, caused by the extra scattering of the Au@AgCSNPs nanorods, which make them more effective in the SERS system.

Due to the excellent catalytic effects, the Au@AgCSNPs can be useful in environmental remediation, particularly in the complete elimination of dyes from different media. As shown by Sinha and Ahmaruzzaman [24], decomposition of organic dyes by using Au@AgCSNPs was established due to the formation of the hydroxyl radicals and superoxide radical anions in aqueous media. This photocatalytic oxidation and degradation process was supported by the valence band holes and the conduction band electrons in the structure of the synthesized Au@AgCSNPs.

The Au@AgCSNPs can also be applied for fast oxidation of carbon monoxide (CO) [19,20], used in developing low temperature fuel cells. In this case, Au@AgCSNPs facilitate the low-temperature CO oxidation process by ameliorating the oxygen (O_2_) adsorption and activation [20]. Accordingly, the oxidation process occurs with high efficiency because the AuNPs adsorb the CO molecules while AgNPs adsorb O_2_.

A variety of methods, most involving sonochemical, laser ablation mediated, and chemical reduction, have been developed for the synthesis of the Au@AgCSNPs, both in aqueous or non-aqueous media [11,18,24,25,26]. The common disadvantages of these synthesis methods are their multi-step character and the requirement of the addition of reducing agents into the reaction mixtures, which can perturb the formation of the Ag shell onto the Au core [17]. Bearing in mind different applications of the bimetallic Au@AgCSNPs in biological systems, the suitability of new methods for the effective and biocompatible synthesis has been recently explored. It was shown that non-equilibrium atmospheric pressure plasmas (APPs) enable the production of pure metallic NPs because of the participation of solvated electrons (e_aq_^−^), reactive oxygen species (ROS), and reactive nitrogen species (RNS) in the reduction process of Au(III) or Ag(I) ions to their metallic forms and nanofluids formation [27,28,29,30,31]. To the best of our knowledge, only two research groups have reported the production of the bimetallic Au-Ag NPs using APPs [32,33] as an alternative to the conventional methods. Shirai and co-workers described the production of CSNPs that consisted of an Au-core and an Ag-shell by electron irradiation of the solution surface using a dual-plasma system based on atmospheric pressure glow discharge (APGD) generated in contact with liquid. The disadvantage of this method was a relatively low production rate of Au@AgNPs, caused by performing the reaction in a stationary mode [32]. Yan and co-workers also found that non-equilibrium APPs could be used to generate the Au core-Ag shell nanostructures in a stationary mode system [33].

It could be expected that the use of a flow-through system for the production of NPs and CSNPs would be more convenient and efficient, with an ability to automate and scale up the process, as compared to the non-flowing systems. For this reason, our research group has demonstrated a new method for the nanostructures production based on the use of the APP microdischarge, *i.e.*, dc-µAPGD generated between an Ar plasma microjet and a flowing liquid cathode [34]. Very recently, using a design of experiments (DoE) approach, this system was optimized for the AuNPs production by monitoring the wavelength at the maximum (λ_max_) of the localized surface plasmon resonance (LSPR) absorption band [35].

The main aim of the current work was to adapt this system to produce Au@AgCSNPs through the plasma-mediated synthesis of the Au core *in situ*, followed by the addition of the Ag nanoshell onto the AuNPs core in the plasma-mediated process. The synthesized Au@AgCSNPs were characterized by ultraviolet-visible absorbance spectrophotometry (UV-Vis), energy-dispersive X-ray spectroscopy (EDS), and transmission electron microscopy (TEM). Additionally, a possible mechanism of the Au@AgCSNPs formation was briefly discussed.

## 2. Results

It was previously shown that the liquid-plasma interface of the flow-through dc-µAPGD system is a rich source of the ROS and the RNS species along with the e_aq_^−^ [34,35,36], which can reduce the Au(III) ions to their metallic form. Indeed, the presence of these reactive species in the liquid-plasma interface of the microdischarge resulted in changing the color of the treated Au working solutions due to reduction of the Au(III) ions. At first, the color of the AuNPs precursor (HAuCl_4_) solutions was yellowish, and was changed to ruby red following the first treatment with dc-µAPGD, which is characteristic of the AuNPs formation. The obtained AuNPs were not agglomerated and no sedimentation was observed, confirming the sufficient stabilization properties of gelatin. The resultant solution containing the gelatin-stabilized AuNPs was immediately mixed with an Ag(I) working solution (at a final concentration of 25 mg·L^−1^) and treated again with dc-μAPGD. After this, the color of the mixture changed to dark yellow, which is characteristic of the AgNPs production. These visual observations were consistent with the synthesis of AuNPs followed by the deposition of an Ag nanoshell onto the AuNPs core, resulting in the formation of Au@AgCSNPs.

**Figure 1:**
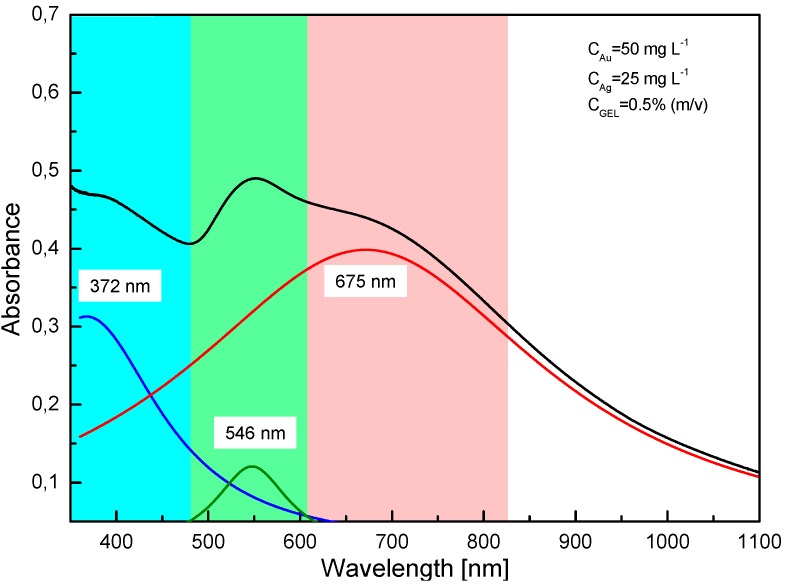
The UV-Vis spectrum of the Au@AgCSNPs. The broad visible absorption spectrum of Au@AgCSNPs (black line) was deconvoluted into three prominent peaks centered at the following wavelength: 372 nm (the absorption plasmon band of the Ag shell—blue line), 546 nm (the absorption plasmon band of the Au core—green line) and 675 nm (the longitudinal plasmon band of the Au core—red line).

**Figure 2:**
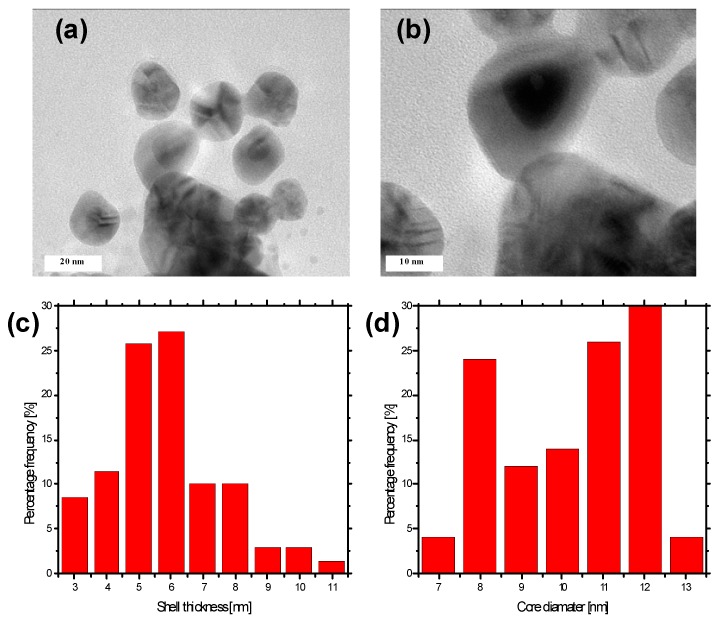
Analysis of the size of the Au@AgCSNPs. (**a**,**b**) Representative TEM micrographs of the Au@AgCSNPs; (**c**) A histogram displaying the size distribution of the shell thickness of the Au@AgCSNPs; (**d**) A histogram showing the size distribution of the core diameter of the Au@AgCSNPs.

**Figure 3:**
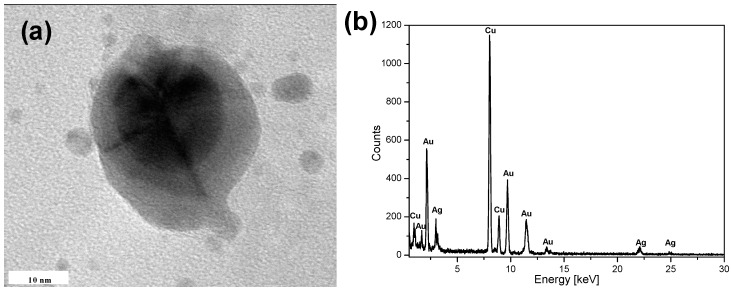
Elemental analysis of the obtained Au@AgCSNPs. (**a**) Representative TEM micrographs of the spherical Au@AgCSNPs; (**b**) EDS analysis of the dark inner core of the Au@AgCSNPs. Numerous large peaks corresponding to Au and Ag are present.

UV-Vis absorption spectrophotometry was used to examine the optical properties of Au@AgCSNPs obtained in the dc-μAPGD system. It is well known that both AuNPs and AgNPs exhibit well determined optical properties dependent on their LSPR bands. The AgNPs show high absorption in the range of 400–450 nm [37] unlike the AuNPs, for which the characteristic LSPR band is within the range of 520–570 nm [38]. In our case, a broad absorption band was observed and deconvoluted using the Gaussian distribution (as depicted in Figure 1) to extract the characteristic LSPRs of the excited electrons. The presence of two LSPR bands situated at 372 nm and 546 nm due to excitation of plasmons on the surface of the NPs confirmed the production of the metallic forms of Ag and Au and provided the evidence that the Au@AgCSNPs were effectively synthesized. Due to a lower concentration of the Ag nanoshell precursor (25 mg·L^−1^) relative to the AuNPs precursor concentration (50 mg·L^−1^), the LSPR band maximum at 372 nm exhibited a lower absorbance value as compared to the absorbance value of the LSPR band maximum at 546 nm for the AuNPs. The plasmon band maximum of the Au@AgCSNPs at 372 nm was blue-shifted and the extent of this was likely related to the thickness of the Ag shell onto the Au core [39]. There was also a third band identified in the spectrum, centered at λ = 675 nm, which was referred to the longitudinal plasmon band of Au core [40]. This band is characteristic of the nanostructures with non-spherical anisotropic shapes, *i.e.*, rodded, triangular, cubed or pentagonal, the presence of which was supported by TEM (Figure 2a,b and Figure 3a).

The metal configuration, the size distribution of the shell thickness, the size distribution of the core diameter, and the shape of the Au@AgCSNPs were analyzed with TEM and EDS. Figure 2a,b show the representative TEM micrographs of the obtained bimetallic Au@AgCSNPs. As can be seen, for all CSNPs, the Ag nanoshell and the AuNPs core are distinguishable as the light and dark regions, respectively, due to the differences in density between the AgNPs and the AuNPs [41]. The approximately spherical Ag nanoshell deposited onto the Au spherical core indicated the production of Au@AgCSNPs. As can be seen, the Au@AgCSNPs are mostly spherical and pentagonal, and well separated from each other. The sufficient separation was likely due to the gelatin used during the AuNPs production as a capping ligand, which prevented the uncontrolled growth of these NPs and their sedimentation. The average thickness of the Ag nanoshell was based on 75 measurements of the CSNPs, whereas the average Au core diameter was based on 50 measurements of these same CSNPs. The thickness of the Ag shell and the Au core were both uniform in size with the average shell size of 5.8 ± 1.8 nm and the average core diameter of 10.2 ± 2.0 nm, respectively. According to Sinha and Ahmaruzzaman [24], no plasmonic properties for AuNPs can be observed when the thickness of the Ag shell is greater than 4.5 times than the Au core diameter.

To confirm that the nanostructures observed in the TEM micrographs indeed consisted of the AuNPs core and the Ag nanoshell, EDS was used to determine the elemental composition of the obtained CSNPs (Figure 3). Numerous large peaks corresponding to the presence of Au were detected in the dark inner regions of the nanostructures (Figure 3a,b) confirming that the core of the Au@AgCSNPs was composed of Au. Some peaks corresponding to Ag could be ascribed to the formation of a thin shell on the core during the simultaneous excitation by the electron beam. The strongest peaks at 8 and 8.9 keV, visible in Figure 3, are due to the Cu sample holder. Thus, all the data reported here indicated that the described dc-µAPGD system could effectively be used for the synthesis of the Au@AgCSNPs.

## 3. Discussion

In the current work, the dc-µAPGD system was adapted for the synthesis of the Au@AgCSNPs. This was accomplished by producing the AuNPs core while operating the system with a flowing liquid cathode (FLC), followed by the synthesis of the Ag nanoshell on the AuNPs after the reversal of the polarity of the electrodes. In the synthesis of the AuNPs core, the AuCl_4_^−^ ions were possibly reduced to the metallic AuNPs through the following reactions: 2AuCl_4_^−^ + 3H_2_O_2_ = 2Au^0^+ 3O_2_ + 6H^+^ + 8Cl^−^ and/or AuCl_4_^−^ + 3e_aq_^−^ = Au^0^ + 4Cl^−^. These reactions are likely because of the presence of the H_2_O_2_ molecules and e_aq_^−^ in the solutions of the flowing liquid cathode for a similar discharge-reaction system, where these solutions are negatively charged [29,34]. The polarity of the electrodes were changed and the Ag ions were possibly reduced to obtain the Ag nanoshell through the following reaction: Ag^+^ + e_aq_^−^ = Ag^0^. The deposition of the Ag nanoshell as the efficiency of the CSNPs production in these conditions was higher. It could be hypothesized that this was related to the reduction potential of Ag and the use of gelatin as the stabilizer during the synthesis of the AuNPs core. The reduction potential of Ag (*E*° = 0.799 V) is lower than that of Au (*E*° = 1.52 V), and so, it could be expected that the reduction of the Ag(I) ions would be more effective operating the system with the flowing liquid anode (FLA) due to the irradiation of the surface of the working solution (the liquid anode) with electrons, which came from the Ar nozzle microjet cathode and contributed to the formation of the Ag nanoshell nuclei onto the AuNPs core [12,32].

In the case of the AuNPs, the synthesized AuNPs could have possibly negative charge, whereas gelatin was positively charged as suggested by Neupane *et al.* [42]. This means that during the AuNPs synthesis, they could be coated with a gelatin shell (and/or products of gelatin hydrolysis), preventing their aggregation and sedimentation. Hence, the AuNPs were rather unavailable to serve as the nucleation sites for the Ag nanoshell deposition when the system was operated with the FLC, *i.e.*, with the positive ions irradiation of the working solution surface. By changing the electrodes polarity, the microdischarge was operated with the FLA and the Ar nozzle microjet cathode was a rich source of free electrons that irradiated the working solution surface. These electrons could destroy the gelatin shell, leaving the AuNPs available for the Ag nanoshell deposition.

In addition to changing the polarity of the electrodes, other parameters of dc-µAPGD had a significant influence on the optical and granulometric properties of the synthesized Au@AgCSNPs. As previously established in our group studying similar dc-µAPGD systems [34,35], the concentration of the AuNPs precursor and the discharge current were the parameters being the most critical for the AuNPs core synthesis. Accordingly, to obtain the AuNPs core of the lowest size, the highest discharge current and the lowest concentration of the AuNPs precursors were needed [35]. The flow rate of the Ar microjet-supporting gas was not significant for the morphology and the optical properties of the obtained CSNPs. All the solutions containing the AuNPs precursor were acidified with HCl to pH~1 to make them conductive. It was confirmed that the NPs could be synthesized from the acidic solutions.

The plasma-mediated synthesis method described in the present work is advantageous over other methods reported in the related literature owing to its rapidness and high-throughput nature. The presented flow-through system for the Au@AgCSNPs synthesis is based on dc-µAPGD generated between the Ar nozzle microjet and the flowing liquid. This system facilitated the generation of the uniform in size and spherical Au@AgCSNPs with the core diameter of 10.2 ± 2.0 nm and the Ag shell thickness of 5.8 ± 1.8 nm. The Au@AgCSNPs of these dimensions have optical properties that are useful in practical applications such as SERS. As compared to previously reported methods for the Au@AgCSNPs synthesis, the system described here has several benefits. Toxic chemical reducing agents are not involved in the process, unlike more traditional methods that use toxic chemical reagents for the reduction of the Au(III) and Ag(I) ions. While other researchers have also used APPs as a replacement of the chemical reducing agents, their systems allowed to perform the synthesis in a stationary mode, unlike the continuous flow mode dc-µAPGD system described here, which limits the synthesis rate of Au@AgCSNPs and requires additional human intervention. The high flow-through rate (90 mL per hour) and possibility to automate the synthesis process means that the system described here can be valuable in the future for the industrial scale up of Au@AgCSNP production.

## 4. Materials and Methods

### 4.1. Reagents and Solutions

The synthesis of Au@AgCSNPs was performed using reagents of analytical grade or better. All aqueous solutions were prepared in re-distilled water. Stock solutions (1000 mg·L^−1^) of Au(III) and Ag(I) were prepared by dissolving solid HAuCl_4_ × 4H_2_O (Avantor Performance Materials, Gliwice, Poland) and AgNO_3_ (Avantor Performance Materials, Gliwice, Poland) in re-distilled water. To prepare the working solutions of Au and Ag at concentrations of 50 mg·L^−1^ and 25 mg·L^−1^, respectively, the respective stock solutions were diluted 20 or 40 times with re-distilled water. Next, the diluted solution of Au was mixed with a gelatin (Rousselot International, Mukwonago, WI, USA) solution to reach its final concentration of 0.5% (*m/v*), and acidified with HCl (Avantor Performance Materials, Gliwice, Poland) to its final concentration of 0.1 mol·L^−1^. Gelatin was added to the Au working solutions as a capping agent, preventing uncontrolled growth and sedimentation of AuNPs. HCl was added to improve conductivity of the working solutions and support the microdischarge between the Ar microjet and the solutions surface. Argon gas (99.996%) was supplied by Messer (Messer, Chorzów, Poland).

### 4.2. Synthesis of the AuNPs Core

**Figure 4:**
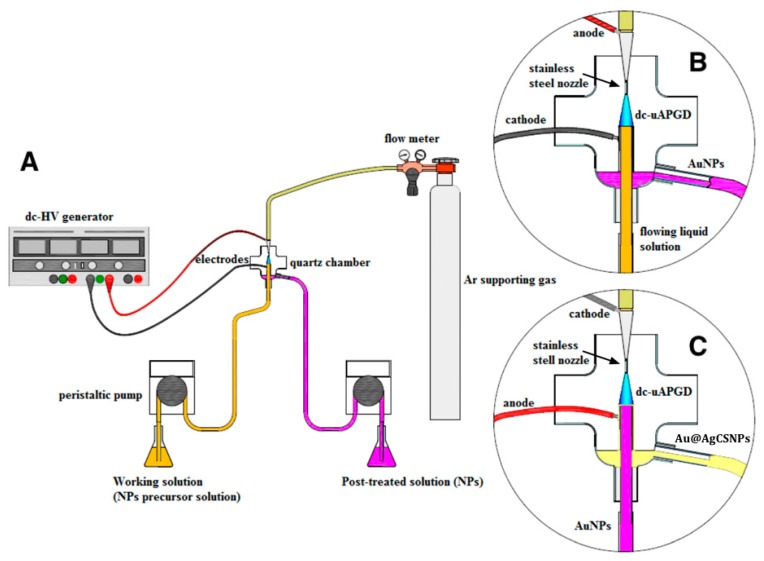
A schematic representation of the dc-µAPGD systems used to obtain the Au@AgCSNPs. (**A**) An overall set up of the dc-µAPGD system; (**B**) A close up of the reactor used to generate the microdischarge between a flowing liquid cathode and an Ar nozzle microjet anode for the AuNPs production; (**C**) A close up of the reactor with the reverse polarity, *i.e.*, using a flowing liquid anode and an Ar nozzle microjet cathode, for the production Au@AgCSNPs.

A miniaturized reactor based on dc-μAPGD was developed to generate the AuNPs core (Figure 4A). The reactor consisted of an Ar nozzle microjet as the anode and an FLC (Figure 4B). The stable microdischarge was sustained between the Ar nozzle microjet anode and the surface of the FLC solution, consisting of HAuCl_4_ × 4H_2_O (the AuNPs precursor), 0.5% (*m/v*) gelatin, and 0.1 mol·L^−1^ HCl. The operating parameters were set as described in the preceding work devoted to the production of AuNPs, having the smallest particle size [35]. The distance between the nozzle and the FLC solution surface was 5.0 mm. The microjet-supporting gas (Ar) was delivered to the system via a stainless steel nozzle (ID 500 μm) at a flow rate of 120 sccm at STP, using a Tylan General (Tylan General Inc., San Diego, CA, USA) mass flow controller (FC-2900) and a Tylan General digital flow meter (RO-28). The Au working solution was introduced to the microdischarge system through a quartz-graphite tube (ID 2.0 mm) at a flow rate of 3 mL·min^−1^ by using a two-channel peristaltic pump (LabCraft Hydris 05, HORIBA Jobin Yvon ICP Instrument, Palaiseau, France). A dc-HV generator (Dora Electronics Equipment, Wrocław, Poland) was used to supply a potential of 1200 V to both electrodes. The discharge current was maintained at 45 mA. A ballast resistor of 10 kΩ (Tyco Electronics, Berwyn, PA, USA) was applied to stabilize the discharge current. After the microdischarge treatment of the Au working solutions, the solutions overflowing the quartz-graphite tube and containing the gelatin stabilized AuNPs were collected into vials.

### 4.3. Synthesis of the AgNPs Nanoshell on the AuNPs Core

The collected solutions containing the gelatin stabilized AuNPs were immediately mixed with a 25 mg·L^−1^ Ag working solution, and such mixtures were again passed through the microdischarge system. In order to add the Ag nanoshell to the AuNPs, the polarity of the electrodes was exchanged. The flowing solutions were positively charged and acted as the FLA. This was done because the efficiency of the core-shell Au-Ag NPs production rate using the FLC was quite low. With the reverse polarity, the Ar nozzle microjet acted as the cathode, hence the surface of the solutions were irradiated with electrons (Figure 4C). Aside from changing the electrode polarity, other components of the reactor were the same as for the synthesis of the AuNPs core. The flow rate of the microjet-supporting gas was 220 sccm at STP. The voltage supplied to electrodes was 1100 V, while the discharge current was maintained at 50 mA. The ballast resistor (10 kΩ) was applied again to stabilize the discharge current. The flow rate of the FLA solutions, being introduced into the reactor, was 3 mL·min^−1^. These operating parameters were set due to the highest stability regime of the microdischarge. After the microdischarge treatment of the solutions containing the gelatin stabilized AuNPs with the added Ag(I) ions, the products of the plasma-mediated synthesis, *i.e.*, the fluids containing the Au@AgCSNPs, were collected into vials and stored for subsequent analysis.

### 4.4. Characterization of the Au@AgCSNPs

In order to confirm the effectiveness of the dc-μAPGD mediated synthesis of the Au@AgCSNPs, the solutions containing CSNPs were analyzed to determinate their optical and granulometric properties.

The optical properties of the Au@AgCSNPs were characterized by UV-Vis absorption spectrophotometry (UV-Vis) with a double-beam UV-VIS Specord 210 Plus (Analytic Jena AG, Jena, Germany). The absorption spectra were recorded 24 h after passing the reaction mixtures (solutions containing the gelatin stabilized AuNPs with the added Ag(I) ions) through the microdischarge reactor. The spectra were acquired in the range from 350 to 1100 nm with a step of 0.1 nm, and a scanning speed of 20 nm·s^−1^. Re-distilled water was used as the reference sample.

The morphology and the size distribution of the Au@AgCSNPs were acquired by transmission electron microscopy (TEM) and energy dispersive spectroscopy (EDS) using a FEI Tecnai G^2^20 X-TWIN instrument (FEI, Hillsboro, OR, USA) supported with an EDAX X-ray microanalyzer (FEI). In order to obtain good-quality resolution of the photomicrographs and to remove non NPs components, the CSNPs were purified with water by three rounds of centrifugation at 12,000 rpm for 10 min in an MPW-350 centrifuge (MPW Medical Instruments, Warsaw, Poland). Finally, 2 drops of a 0.1 mol·L^−1^ NaOH solution were added to the CSNPs washed in water to facilitate their re-suspension. The samples for the TEM and EDS measurements were prepared by putting a drop of the purified solutions onto a copper mesh grid and allowing it to dry.

## Abbreviations

Au@AgCSNPs	Au-Ag core-shell nanoparticles
AuNPs	gold nanoparticles
UV-Vis	ultraviolet-visible
TEM	transmission electron microscopy
EDS	energy-dispersive X-ray spectroscopy
SERS	surface-enhance Raman scattering
APPs	atmospheric pressure plasmas
ROS	reactive oxygen species
RNS	reactive nitrogen species
DoE	design of Experiments
LSPR	localized surface plasmon resonance
FLC	flowing liquid cathode
FLA	flowing liquid anode
